# Assessment of Cardiopulmonary Resuscitation Knowledge Among Physicians in the Pediatrics Department of an Urban Tertiary Referral Hospital in Ethiopia: A Cross-Sectional Study

**DOI:** 10.1155/2024/8815197

**Published:** 2024-10-28

**Authors:** Muluwork Tefera Dinberu, Dagmawi Hailu Yemane

**Affiliations:** Department of Pediatrics and Child Health, Addis Ababa University, Addis Ababa, Ethiopia

**Keywords:** average, knowledge, poor, resuscitation

## Abstract

**Background:** Early recognition of cardiac arrest and prompt start of cardiopulmonary resuscitation (CPR) boost survival rates and reduce postarrest consequences. Little information is available about the fundamental CPR knowledge of healthcare workers who work with children in Ethiopia.

**Methods:** All physicians, regardless of seniority, participated in this cross-sectional survey from June to August 2022. They received a structured survey that was modified from the American Heart Association (AHA) Basic Life Support (BLS) test which was made up of 10 questions about participants' job experience and 25 multiple-choice CPR knowledge questions. Data analysis was done using a multinomial logistic regression test with a *p* value of 0.05.

**Result:** One hundred sixty-eight doctors with various levels of seniority participated in this study. The participants included a male-to-female ratio of 1.3:1, a median age of 28 years, 92 (57.9%) male participants, and 124 (78%) participants with less than 5 years of clinical experience. Ninety-seven participants, or 61%, had scored less than 75% whereas 13 (8.2%), participants, had good knowledge that is scoring above 75%. Participants who had training in CPR within the previous year showed significantly higher levels of knowledge than those who hadn't. Even though 90% of the participants claimed to have CPR knowledge, the majority of participants were found not to have below 75%.

**Conclusion:** The study concludes that while many doctors believe they have adequate CPR knowledge, actual knowledge levels are insufficient. Staff should undergo regular certification and assessments to ensure they retain their resuscitation knowledge. This ongoing evaluation is crucial for maintaining high standards of care and preparedness in emergencies.

## 1. Background

A patient with low oxygen levels and a stopped heart needs to have cardiopulmonary resuscitation (CPR). CPR should be started as soon as possible to avoid multiple organ failure and death. Studies have shown that children have better neurologic outcomes than adults, as evidenced by the survival rate of children after CPR [1].

Due to a lack of proper resources and training, doing CPR has proven to be a difficult task for healthcare professionals in Africa, especially in rural hospitals. Although children who received CPR had better post-CPR outcomes, the quality of CPR was reduced in countries with inadequate resources [2, 3]. Lack of basic CPR knowledge has an impact on the outcome of resuscitation, delays in the time of resuscitation commencement, and the length of time required for resuscitation [4–7].

Studies in several developing countries have shown varying degrees of knowledge among healthcare workers some of it are as follows.

A survey was done in Nigeria that included 140 doctors, 140 nurses, and 140 dentists. Only one respondent, a dentist, achieved a perfect score of 100%. Overall, 58.3% of the participants had inadequate knowledge. This is far away from the standard. The average scores for doctors, dentists, and nurses were 53.5%, 43.3%, and 38.4%, respectively. Multivariate analysis identified doctors, individuals with prior Basic Life Support (BLS) training, and those who graduated 6–10 years ago as significant predictors of adequate knowledge [8].

While a study conducted in South Africa on 245 academic staff revealed only 22.5% of them scored above 75% [9]. Another study conducted in Malaysia found a statistically significant association (*p* < 0.001) between the duration of clinical practice and the confidence level of physicians. Furthermore, 77.1% of respondents recommended that BLS should be re-certified every 2 years [10].

Furthermore, a study conducted in Tanzania involving 350 physicians working in an urban tertiary hospital found that 57 physicians (16%) scored above the local passing mark of 50%, while 13 physicians (4%) scored above 75% [11]. Additionally, a study conducted among health professionals in Nepal revealed that out of 145 complete responses, only 9% were able to correctly identify the sequence of actions from a given set of 7 CPR steps [12].

On the other hand, a South African study involving 173 doctors demonstrated notably higher knowledge levels. Specifically, 61% of these doctors exhibited good knowledge of pediatrics CPR [13].

In general, cardiac arrest can occur both in hospitals and outside of hospitals. However, in Ethiopia, prehospital care is not well developed. The majority of cardiac arrests are typically observed in an emergency room, operating room, and intensive care unit (ICU) because critically unwell pediatric patients frequently visit these settings [14]. The most frequent cause of cardiac arrest in hospitals among children is respiratory failure. The outcome of in-hospital cardiac arrest is currently better in infants than in those who are older, according to the American Heart Association's (AHA) national registry of CPR [15].

In a study conducted in Pakistan [16] on CPR knowledge, out of 724 respondents, 601 (83%) had poor knowledge of CPR, while 123 (17%) were doctors with good knowledge. Notably, doctors who received formal CPR training had significantly better knowledge (20.17%) compared to those who did not receive any training (4.69%). While in an Egyptian study [17] among the 60 junior doctors who participated in the study, only 31.7% had adequate knowledge of CPR. Junior doctors and medical students with prior CPR training showed significantly better knowledge compared to those without any previous training.

In the majority of Ethiopian studies [18, 19], CPR knowledge is poor. The primary reason cited is that BLS training is not widely provided [18]. Additionally, schools do not include CPR training in their curriculum, and even when it is included, it is not taught by appropriately trained individuals [18]. The possible reason for poor prehospital care is an inadequate number of ambulances and Emergency Medical Technicians (EMTs). Unfortunately, these few ambulances are underutilized by the population due to a lack of awareness and long waiting times [20, 21].

The pediatric emergency medicine specialty was introduced at our hospital in 2010 [22]. However, since its inception, there has been no performance evaluation of the healthcare professionals involved. Consequently, this study assessed the knowledge of CPR among physicians in the pediatric department at Tikur Anbessa Hospital.

## 2. Methods

### 2.1. Study Area and Period

The study was conducted between June 2022 and August 2022 at Tikur Anbessa Specialized Hospital (TASH), located in Addis Ababa, Ethiopia which is the largest referral hospital in Ethiopia. TASH is a national referral center located in Addis Ababa, Ethiopia with a bed capacity of 700 beds. One of the departments at TASH the pediatric department serves as a specialized referral department. It accepts patients from all over the country. The pediatric department has 189 beds and admits approximately 9500 to 10,000 patients annually. The department provides outpatient services to around 63,000 patients.

Ethiopia is located in the Horn of Africa. It has an estimated population of 126.5 million people and is considered a low-income country.

### 2.2. Study Design

The study was cross-sectional.

### 2.3. Source Population

During the period of the study, the department was staffed by a total of 210 doctors, encompassing both undergraduate and graduate students. This cohort included Interns, Residents, Specialists, and Subspecialists.

• Interns are medical graduates who have concluded their medical school education. They typically undertake a 1-year rotation in major departments such as internal medicine, pediatrics, gynecology, and surgical departments.
• Pediatrics Residents are individuals who, having completed medical school, are now engaged in a 3-year residency program specifically within the Department of Pediatrics and Child Health. This residency provides them with practical experience and specialized training in pediatric medicine.
• Specialists are physicians who have completed their residency and are now practicing as pediatricians. Their focus is on delivering specialized care to children and adolescents.
• Sub specialists have undergone further specialized training in specific pediatric disciplines. For instance, they may specialize in areas such as pediatric cardiology, pediatric oncology, or pediatric neurology.

### 2.4. Study Population and Sampling Method

The study aimed to include all 210 physicians within the department, encompassing a diverse group of medical professionals such as general pediatricians, pediatricians with various sub specialties, residents, and interns who could potentially be involved in pediatric CPR. Efforts were made to approach each of these individuals on their willingness to participate in the study. Ultimately, 168 physicians were accessible and expressed their willingness to take part in the research. This resulted in a substantial response rate of 80%, reflecting a high level of engagement and interest among the targeted medical staff. In the current study, nurses were not included because a parallel study was being conducted to assess their knowledge. This separate study focused specifically on evaluating the knowledge of nurses regarding pediatric CPR.

### 2.5. Study Variables

To evaluate the CPR knowledge of the clinical team at the pediatric emergency unit we calculated from the knowledge questions section of an administered questionnaire as the dependent variable. Concurrently, we identified several independent variables that could potentially influence the participants' CPR knowledge. These encompassed the clinical team's level of specialization, their cumulative years of clinical experience, their self-perceived understanding of resuscitation procedures, their practical experience in resuscitating patients, and information about their formal CPR training, including the timing of such training, and training on the use a defibrillator.

### 2.6. Data Collection Techniques

Each participant completed a structured, self-administered questionnaire that collected information on demographics, clinical experience, level of training, and employment history. The questionnaire also included details about their certification from a BLS course. Notably, the pass mark for the BLS test was modified from the AHA standard of 85%–75%, to align with our institution's pass mark criteria [11]. In this study, every question carries the same weight, and the final score is converted into a percentage for easier interpretation and comparison. Confidentiality was ensured by avoiding personal identification on the questionnaire.

### 2.7. Data Analysis and Presentation

After entering the data into SPSS version 25, descriptive data analysis was performed. The results were given as numbers, percentages, and odds ratios after bivariate and multivariable logistic regressions were done. The *p* values were taken as significant at below 0.05 or with 95% confidence intervals.

CPR knowledge was stratified into three distinct categories based on the participants' scores:

• Those scoring less than 50% were categorized as having poor knowledge.
• Participants with scores ranging from 50% to 74% were classified as having average knowledge.
• Lastly, those who scored greater than 74% were deemed to have good knowledge.

This classification system was based on a Tanzanian study [11] which also used the AHA-BLS test and classified the knowledge score using the Muhimbili University of Health and Allied Sciences (MUHAS) minimum passing criterion of 50%. Additionally, the minimal passing score of 75% set by the UK Resuscitation Council was also taken into consideration. This approach provides a comprehensive assessment of the participants' CPR knowledge levels.

## 3. Results

### 3.1. Demographics

A total of 168 participants from the pediatrics and child health departments were included in the study. The majority of the participants was male, and around the age of 28 as most of them were either residents (56.5%) or interns (31.5%) with less than 5 years of clinical experience (79.2%) (Table 1).

Of the participants, 110 (65.5%) had average CPR knowledge, and only 15 (8.9%) participants with good CPR knowledge of which 7 were Interns and 8 were Residents. Most of the participants (73.8%) had previously tried to resuscitate a child, of which 29 (23.4%) had poor CPR knowledge. A majority of the participants also had formal CPR training, including 86.7% of those with good CPR knowledge. Furthermore, 72.6% of those who had CPR training less than a year prior had average CPR knowledge, which is slightly higher than those with training less than 5 years prior (68.9%). In addition, despite many participants who reported knowing what a defibrillator is, only nearly half reported having one in the department. Moreover, 69.9% did not have training on how to use one (Table 2).

### 3.2. Level of Specialization, CPR Training, and Defibrillator Knowledge

**Figure 1:**
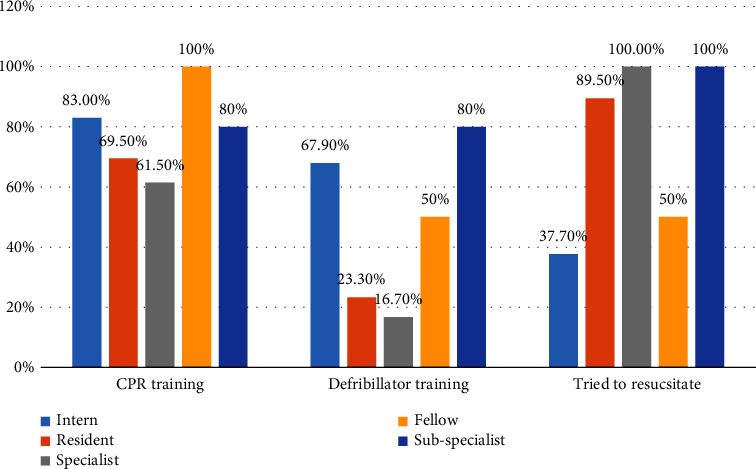
Description of participants by level of specialization for the CPR knowledge assessment at Tikur Anbessa Specialized Hospital, Addis Ababa, Ethiopia 2023.

Among the participants, Specialists had the lowest proportion of CPR training (61.5%) and Defibrillator training (16.7%) while all specialists had experience resuscitating a child. Furthermore, a larger proportion of Interns had CPR (83%) and Defibrillator (67.9%) training compared to Residents 69.5% and 23.3%, respectively (Figure 1).

### 3.3. CPR Knowledge

After adjusting for possible confounders using multivariable logistic regressions, it was found that Residents (OR = 0.27, *p*=0.008) and Specialists (OR = 0.24, *p*=0.047) were found to be less likely to have average CPR knowledge relative to Interns. Likewise, Residents (OR = 0.22, *p*=0.027) were less likely to have good CPR knowledge relative to Interns. Furthermore, participants who reported that they knew how to resuscitate were 5.78 times more likely (*p*=0.017) to have average CPR knowledge. While those with formal CPR training were 4.05 times more likely (*p*=0.001) to have average CPR knowledge and 6.21 times more likely (*p*=0.026) to have good CPR knowledge. In addition, of those with formal CPR training, those who did the training less than 5 years ago were less likely (OR = 0.14, *p*=0.025) to have good CPR knowledge relative to those who did the training less than a year ago (Table 3).

## 4. Discussion

As far as we are aware, this is the first study to evaluate the CPR knowledge of doctors working at Tikur Anbessa Hospital's pediatrics department. Health workers working at teaching hospitals like ours are frequently believed to have better BLS/CPR knowledge, but this was not the case as only around 9% had good CPR knowledge.

The participants' median age was 28 which are younger than a study conducted in Tanzania (35 years old) [11]. This is most likely because our study had predominantly included Interns and Residents, as opposed to the Tanzanian study which included Nurses and health attendants. Male participants made up the majority of 103 (61.3%) participants, in contrast to a study conducted in Malaysia [10] which was predominately female (60%). In our survey, 110 participants, or 65.5%, had average knowledge, whereas 15 participants, or 8.9%, had good knowledge. This is greater than the Tanzania study [11] where 13 (4%) of the participants had good knowledge, but lower than the one done in South Africa [9] where 245 (22.5%) had good knowledge and also lower than the Pakistan and Egyptian study 17% and 31.7% respectively [16, 17]. This could be explained by the fact that the Tanzanian study had significant numbers of non-physicians and in the South African study, the participants received regular CPR training in our study, we relied solely on self-administered questionnaires, which presents a significant limitation in accurately assessing the participants' knowledge. A study was done in Nigeria on medical students [13] working in one of the teaching hospitals had 97.1% poor CPR knowledge despite having CPR training. This was most likely due to the lack of hands-on training including simulation in their program.

Participants in this study who had less than 5 years of work experience had a larger percentage of good knowledge scores than those who had between five and 10 years and more than 5 years of experience. The reason for this disparity is most likely that the residents and interns recently completed BLS/CPR training. Unlike our study, a Malaysian [10] investigation found that doctors with more work experience performed better than those with less work experience. This difference may be explained by the fact that in the Malaysian study, the performance of CPR was evaluated using more than just theoretical knowledge.

Participants who had received BLS/CPR training in the past performed statistically significantly much better on knowledge tests than those who had not. This is comparable to the research Garcia [8] conducted. This suggests that frequent BLS/CPR teaching is required to ensure adequate knowledge. Furthermore, individuals who finished their training less than a year prior were more likely to demonstrate good CPR knowledge compared to those who took training longer prior.

Interns' and Residents knowledge was found to be superior to Pediatricians' and Pediatric sub specialists. The reason for this is that interns and residents underwent BLS/CPR training before graduating from medical school, and residents rotated through the emergency unit during their first year of residency. This is in line with the Tanzanian study [11]. The lack of regular training, updating courses, and certification in BLS/CPR for physicians working in pediatric departments is a significant issue. This gap in continuous education and certification means that many physicians may not be up-to-date with the latest guidelines and techniques, potentially impacting the quality of care provided during pediatric CPR.

The findings show that despite the clinical team knowing what a defibrillator is a majority of them report having no training with defibrillator and not being available in the department. This could be due to the lack of hands-on experience among most participants in manipulating the Automated External Defibrillator (AED) which could be attributed to the fact that only one AED was available for training. This AED was primarily used for demonstration purposes during the defibrillator training. It is crucial to address this gap in knowledge by providing hands-on practice with AEDs during training sessions. Familiarity with AED operation and its availability in the emergency department is essential for effective response during cardiac emergencies.

### 4.1. Limitations of the Study

One of the limitations of the study is that only theoretical knowledge in the form of a questionnaire was assessed; no evaluation of practical knowledge was done. This can bring to light individuals who possess solid information but may not perform properly.

Second, the survey was self-administered so that respondents may review the literature. This might cause a bias in the responses to the questionnaire. The study has however shown that the department's health staff has average knowledge of BLS/CPR.

Thirdly, while the survey focused solely on the CPR knowledge of physicians working in the pediatric department, it is crucial to recognize that other clinical team members also play a role in patient resuscitation. To gain a more comprehensive understanding, we should consider this study alongside others that explore CPR knowledge among different healthcare providers.

## 5. Conclusion

Since 2010, BLS/CPR training has been provided to healthcare professionals working in pediatric emergency settings. This study was conducted to evaluate the impact of both preservice and in-service training on these professionals. By assessing the effectiveness of the training programs, the study aimed to determine how well they prepare healthcare workers for pediatric emergencies and identify areas for improvement in ongoing education and certification efforts. The assessment of pediatric CPR knowledge among the physicians in the department revealed significant gaps. The findings indicate that the current level of understanding and proficiency in pediatric CPR is insufficient. Consequently, there is a clear and urgent need for comprehensive training programs to be implemented for all healthcare workers in the unit. This training should aim to enhance their knowledge and ensure they are well-prepared to handle pediatric emergencies effectively.

## 6. Recommendations

For enhancing the quality of CPR performance, it is crucial to have access to necessary equipment such as AEDs, bag-valve masks (Ambu bags), and monitoring devices. It is important to perform regular maintenance and checks on this equipment to ensure they function properly during emergencies.

Healthcare professionals should receive regular and ongoing BLS training. This training should encompass key skills like chest compressions, airway management, and hands-on training on AEDs. These could be one of the requirements for licensing.

Simulation sessions should be conducted regularly, ideally every 2–3 months, to mimic real-life scenarios. These simulations provide healthcare providers with the opportunity to practice teamwork, communication, and critical decision-making during emergencies. It is also beneficial to include pediatric scenarios to tackle the unique challenges involved in caring for children.

After each simulation or real-life event, it is recommended to conduct debriefing sessions. These sessions can be used to discuss what was done well and identify areas that need improvement. This approach ensures continuous learning and improvement in the quality of CPR performance.

## Figures and Tables

**Table 1:** Demographic characteristics of participants for the CPR knowledge assessment at Tikur Anbessa Specialized Hospital, Addis Ababa, Ethiopia, 2023.

Characteristics		*n* (%)
Age	Mean ± SD, median	28.4 ± 4.5, 28

Sex	Male	103 (61.3)
	Female	65 (38.7)

Level of specialization	Intern	53 (31.5)
	Resident	95 (56.5)
	Specialist	13 (7.7)
	Fellow	2 (1.2)
	Sub-specialist	5 (3)

Years of clinical experience	Less than 5 years	133 (79.2)
	5–10 years	29 (17.3)
	More than 10 years	6 (3.6)

**Table 2:** CPR knowledge characteristics of participants for the CPR knowledge assessment at Tikur Anbessa Specialized Hospital, Addis Ababa, Ethiopia, 2023.

Characteristics			Knowledge		
		*n* (%)	Poor	Average	Good
Total		168 (100)	43 (25.6)	110 (65.5)	15 (8.9)

Sex	Male	103 (61.3)	28 (27.2)	66 (64.1)	9 (8.7)
	Female	65 (38.7)	15 (23.1)	44 (67.7)	6 (9.2)

Level of specialization	Intern	53 (31.5)	6 (11.3)	40 (75.5)	7 (13.2)
	Resident	95 (56.5)	31 (32.6)	56 (59)	8 (8.4)
	Specialist	13 (7.7)	5 (38.5)	8 (61.5)	0
	Fellow	2 (1.2)	0	2 (100)	0
	Sub-specialist	5 (3)	1 (20)	4 (80)	0

Years of clinical experience	Less than 5 years	133 (79.2)	35 (26.3)	83 (62.4)	15 (11.3)
	5–10 years	29 (17.3)	7 (24.1)	22 (75.9)	0
	More than 10 years	6 (3.6)	1 (16.7)	5 (83.3)	0

Tried to resuscitate a child	Yes	124 (73.8)	29 (23.4)	84 (67.7)	11 (8.9)
	No	44 (26.2)	14 (31.8)	26 (59.1)	4 (9.1)

Formal CPR training	Yes	124 (73.8)	22 (17.7)	89 (71.8)	13 (10.5)
	No	44 (26.2)	21 (47.7)	21 (47.7)	2 (4.6)

CPR training time	Less than a year ago	73 (58.9)	9 (12.3)	53 (72.6)	11 (15.1)
	Less than 5 years ago	45 (36.3)	12 (26.7)	31 (68.9)	2 (4.4)
	More than 5 years ago	6 (4.8)	1 (16.7)	5 (83.3)	0

Do you know how to resuscitate a dying person?	Yes	157 (93.5)	37 (23.6)	107 (68.1)	13 (8.3)
	No	11 (6.5)	6 (54.5)	3 (27.3)	2 (18.2)

Know what a defibrillator is	Yes	152 (97.4)	34 (22.4)	105 (69.1)	13 (8.5)
	No	4 (2.6)	2 (50)	2 (50)	0

Training on how to use a defibrillator	Yes	47 (30.1)	12 (25.6)	30 (63.8)	5 (10.6)
	No	109 (69.9)	24 (22.1)	77 (70.6)	8 (7.3)

Defibrillator available in department	Yes	73 (47.1)	15 (20.6)	50 (68.5)	8 (10.9)
	No	82 (52.9)	21 (25.6)	56 (68.3)	5 (6.1)

**Table 3:** Odds ratios of good CPR knowledge of participants for the CPR knowledge assessment at Tikur Anbessa Specialized Hospital, Addis Ababa, Ethiopia, 2023.

Characteristics		Good CPR knowledge	
		OR	*p* value
Level of specialization	Intern	1	
	Resident	0.22	**0.027**
	Specialist	—	—
	Fellow	0.06	0.995
	Sub-specialist	—	—

Years of clinical experience	Less than 5 years	1	
	5–10 years	—	—
	More than 10 years	—	—

Do you know how to resuscitate a dying person?	Yes	1.05	0.952
	No	1	

Tried to resuscitate a child	Yes	1.33	0.672
	No	1	

Formal CPR training	Yes	6.21	**0.026**
	No	1	

CPR training time	Less than a year ago	1	
	Less than 5 years ago	0.14	**0.025**
	More than 5 years ago	—	—

Training on how to use a defibrillator	Yes	1.25	0.739
	No	1	

*Note:* Bold values represent statistically significant at a *p* value less than 0.05.

## Data Availability

The datasets used and/or analyzed during the current study are available from the corresponding author on reasonable request.

## Nomenclature

ACD: Active compression-decompression
AED: Automated External Defibrillator
AHA: American Heart Association
PCA: Pediatric cardiac arrest
BLS: Basic Life Support
CPR: Cardiopulmonary resuscitation
HIE: Hypoxic-ischemic encephalopathy
IHCA: In-hospital cardiac arrest
MET: Medical emergency team
NSE: Neuron-specific enolase
OHCA: Out of hospital cardiac arrest
TASH: Tikur Anbessa Specialized Hospital
